# Investigating layer-by-layer chitosan-dextran sulfate-coated mesoporous silica as a pH-sensitive drug delivery system

**DOI:** 10.1007/s10856-024-06797-9

**Published:** 2024-06-17

**Authors:** Mohammad Reza Hooshyar, Shahram Raygan, Rouhollah Mehdinavaz aghdam

**Affiliations:** 1 Synthesis and Extraction of Materials Lab., School of Metallurgy and Materials Engineering, College of Engineering, University of Tehran, P.O. Box 11155-4563, Tehran, Iran; 2 Biomaterials Lab., School of Metallurgy and Materials Engineering, College of Engineering, University of Tehran, P.O. Box 11155-4563, Tehran, Iran

## Abstract

Mesoporous silica nanoparticles (MSNPs) coated by chitosan (CS) were shown to be a proper candidate as a carrier for drug delivery purposes. However, choosing the suitable drug-containing complexes to be applied on MSNPs-CS is of much greater importance to evaluate the possible candidate for an efficient combination of cell viability, drug release kinetics, and atherosclerosis prevention. In this regard, this study concentrates on the synthesis and assessment of coated MSNPs-CS designed for drug delivery purposes. The MSNPs are coated with polyelectrolyte complexes (PEC) composed of CS and dextran sulfate (MSNPs-CS-DX), serving as a versatile drug carrier with favorable biological characteristics. CS-DX is applied to MSNPs without requiring complex or multi-step synthesis procedures. Rosuvastatin, a cholesterol-lowering medication, is chosen for its therapeutic relevance. Additionally, CS-DX is found to relatively impede the uptake of low-density lipoproteins (LDLs) by macrophages, enhancing their potential therapeutic utility. FTIR pattern, FESEM, and TEM images prove MSNPs-CS-DX formation. DLS measurement demonstrates the average particle size of 110 nm for MSNPs, with the combined thickness of CS and DX layers ranging from 10 to 15 nm. BET test is carried out to evaluate the pore size and porosity of structure, showing outstanding results that cause an entrapment efficiency of 57% for MSNPs-CS-DX. Furthermore, the findings demonstrate the pH sensitivity of MSNPs-CS-DX on drug release kinetics. Notably, the CS-DX layer exhibits a significant enhancement in cell viability of human umbilical vein endothelial cells (HUVEC) by approximately 24% within a 24 h timeframe compared to MSNPs lacking CS-DX.

**Graphical Abstract:**

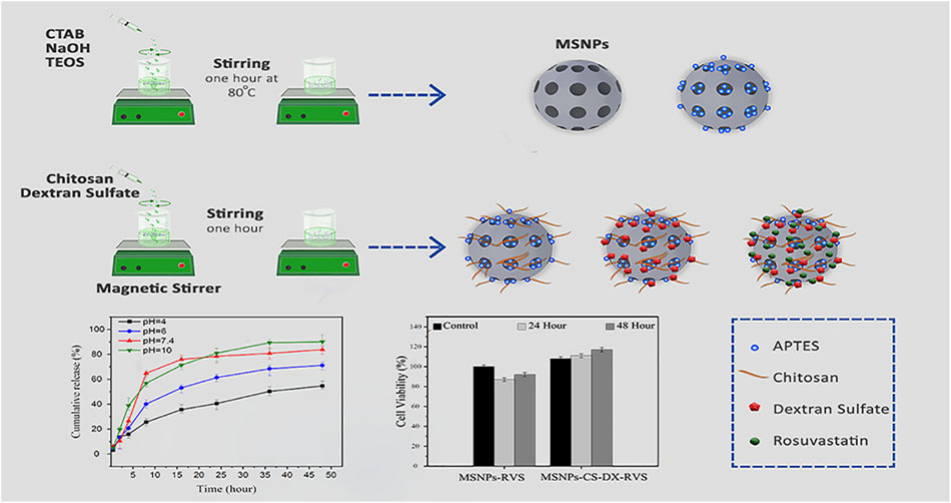

## Introduction

In recent years, mesoporous silica has emerged as a prominent candidate for drug delivery due to its non-toxicity, biocompatibility, and ease of functionalization. Mesoporous silica nanoparticles (MSNPs) have received a great deal of scrutiny in various studies, typically synthesized through methods such as sol-gel, hydrothermal, chemical etching, and template-assisted techniques [1–5]. The material in question displays a wide range of advantageous properties for drug delivery systems, including adjustable pore size, extensive surface area, high drug-loading capacity, and the ability to effectively immobilize therapeutic compounds. While MSNPs are employed in various drug delivery systems, there is a notable scarcity of investigations specifically targeting atherosclerosis treatment. Atherosclerosis stands as the foremost cause of heart attacks, characterized by chronic inflammation within the arterial walls. This inflammatory environment prompts the cellular release of free radicals, leading to the oxidation of low-density lipoproteins (LDLs). Elevated levels of LDL cholesterol in the bloodstream are linked to increased risks of atherosclerosis, heart attack, and stroke. Recent studies have demonstrated that silica nanoparticles exhibit superior antioxidant properties compared to other nanomaterials, including iron oxide, gold, titanium, and platinum [6–8]. These antioxidant properties play a crucial role in the treatment of heart-related diseases. Furthermore, through chemical functionalization of the mesoporous structure by adjusting the pore diameter or incorporating nanocapsules to regulate timing, precise control over drug release can be attained. Studies have indicated that the biological properties of mesoporous silica can be enhanced through the incorporation of chitosan (CS). Liang et al., [9]. Anitha et al., [10–12]. Using (3-Aminopropyl) triethoxysilane (APTES) can not only reduce the necessary charge for electrostatic bonding between MSNPs and CS but also enhance the absorption capabilities of CS and prime it for subsequent synthesis routes. APTES prevents MNSPS from oxidation, resulting in its employment in these systems [13, 14].

CS, derived from natural polysaccharides comprising β-1,4-linked glucosamine residues, is a biopolymer known for its diverse biological attributes, including mucoadhesive and antibacterial properties. Additionally, CS has been extensively utilized in various formulations for drug delivery. Heidari et al., [15, 16]. This biocompatible material is capable of forming complexes with DNA and polyanionic polymers. Moreover, CS reduces blood cholesterol levels and inhibits the body’s absorption of dietary cholesterol [17, 18].

Dextran sulfate (DX), composed of α-1,6-d-glucopyranose polysaccharide units, exhibits hydrophilic, biodegradable, and biocompatible characteristics. Numerous studies have documented the anticoagulant, antiviral, and cholesterol-lowering properties of DX. Davis, McLister [19–21]. It consists of multiple sulfate groups, imparting a negative charge that facilitates the synthesis of CS and DX in a combination layered manner. Additionally, due to the positively charged molecule (apolipoprotein B) in their structure, LDLs can bind to DX, preventing their absorption by macrophages and inhibiting the formation of new plaques. Furthermore, DX can bind to the scavenger receptor A (SR-A), thereby enhancing both its binding capacity and the local delivery of the drug by the carrier to the site of plaque formation. You et al., [22, 23].

pH sensitivity is of significant importance for drug delivery systems, particularly considering the fluctuating local pH in target sites and departure from neutral environments. This principle holds even for complications such as heart vessel blockage. In areas where platelet formation occurs, the presence of macrophages leads to a progression towards acidification of the environment. The high lactate concentration, possibly stemming from macrophage accumulation, contributes to the acidic milieu [24]. Based on the literature, CS-DX would serve as a pH-sensitive complex.

Rosuvastatin (RVS) stands as one of the most widely prescribed cholesterol-reducing medications worldwide [25]. Its efficacy in reducing the risk of cardiovascular disease has been demonstrated in primary and secondary prevention settings [26]. Hence, RVS was chosen as the therapeutic agent for this research.

While combination layers of CS-DX on diverse materials have been extensively studied, the synthesis and investigation of MSNPs featuring CS-DX layers for drug delivery purposes remain unexplored [8, 27]. Because the surface of MSNPs is amenable to functionalization, an electrostatic physical bond between silica and the initial layer, CS, would be formed. Considering the aforementioned details, MSNPs-CS-DX can be found to be a pH-sensitive nanocarrier. Hence, investigating the MSNPs-CS-DX as a carrier for drug delivery purposes can shed more light on this field of research.

The primary objective of this study is to develop novel compounds using a straightforward and innovative approach across multiple stages for drug delivery purposes. In this regard, layered MSNPs containing CS and DX are synthesized to assess their capability for loading and releasing RVS. The selection of this material is based on the observed property of CS in reducing cholesterol absorption, aimed at alleviating coronary artery occlusion. Additionally, the surface charge of DX can hinder the formation of new vascular platelets. Therefore, utilizing the synthesized RVS delivery drug may serve the dual purpose of preventing and treating coronary heart disease.

## Materials and methods

### Materials

Tetraethyl orthosilicate (TEOS), cetyltrimethylammonium bromide (CTAB), 3-aminopropyltriethoxysilane (APTES), the sodium salt of dextran sulfate (mol wt > 500 KDa), low molecular weight CS (degree of deacetylation, DD > 75%) were from Sigma–Aldrich. RVS was purchased from Hakim Farayand Chemical. Ethanol, methyl alcohol, anhydrous toluene, and all other chemicals used were from Sigma-Aldrich with high purity. Human umbilical vein endothelial cells (HUVECs) were from the National Cell Bank of the Pasteur Institute of Iran.

### Methods

#### Preparation of mesoporous silica

To prepare the solution, 1 g of CTAB was added to 480 mL of distilled water. NaOH solution (3.5 mL, 2 M) was introduced to the CTAB-containing solution at the temperature of 80 °C. Over 15 min, 5 mL of TEOS was gradually added dropwise to the stirred solution. Following this, the mixture was stirred for 2 h until a white precipitate appeared, and the precipitate was filtered, washed with methanol and distilled water twice, and dried at room temperature. To remove the CTAB template, 2 g of the powder was dispersed in a solution comprising 200 mL of methanol and 2 mL of HCl (32%) for 6 h. The synthesized MSNPs were filtered and washed with methanol and distilled water. Subsequently, MSNPs were dried under vacuum at room temperature to obtain pure mesoporous silica devoid of CTAB surfactant. To produce functionalized mesoporous silica, 1.5 g of mesoporous silica was refluxed in 100 mL of anhydrous toluene containing 0.3 mL of APTES [28, 29].

#### Coating of mesoporous silica nanoparticles by chitosan and dextran sulfate

The preparation of multilayer MSNPs-CS-DX requires stirring and centrifuging processes. Initially, MSNPs were dispersed in 25 mL of distilled water (2.0 wt%). Subsequently, 25 mL of CS solution with a concentration of 2 mg/mL was added to the MSNPs solution and stirred for 1 h. The mixture was then centrifuged at 12,500 rpm for 3 min, followed by washing with methanol and distilled water, and re-dispersed in a 0.5 M NaCl solution. A DX solution was prepared in 25 mL of NaCl solution with a concentration of 2 mg/mL and stirred for 1 h at 12,500 rpm. Afterward, the solution was centrifuged and washed with methanol and distilled water. This procedure was repeated twice. Ariga et al., [30–32].

#### Rosuvastatin loading

A total of 0.2 mg of multilayer MSNPs-CS-DX was added to 25 mL of a 2 mg/mL RVS solution in distilled water. The resulting suspension was then added to 30 mL of PBS solution. Following 12 h of stirring under dark conditions, the nanocarriers were separated by centrifugation at 12,500 rpm for 3 min. The supernatant was utilized to determine the RVS loading using a UV–vis spectrophotometer. Ariga et al., [30].

### Characterization

Transmission electron microscopy (TEM, Philips CM200, Netherlands) and dynamic light scattering (DLS; Horiba sz100 Japan) were used to evaluate the mesoporous structure and morphology of the nanoparticles. A Field emission scanning electron microscope (FE-SEM, Hitachi 4160) with EDS analysis was used to observe and analyze the morphology and size of the nanocarriers. Also, the size of the particles was checked from the TEM and FE-SEM images by the Digimizer software. BET analysis was performed at −196 °C to measure surface area, pore diameter, and distribution (Belsorp, the sample was degassed under vacuum conditions at a temperature of 200 °C for 10 h). The powder samples were further evaluated by Fourier transform infrared spectrophotometry (FTIR, Nicolet American) and X-ray diffraction (XRD, Philips 1730 PW, Cu-kα, the step size was 0.04°, and the scan rate was 4°/min). The loading and releasing of the drug in solution were measured by UV–vis spectrophotometer (Biobase BK-UV1200). The fluorescence spectrum was measured with a Perkin Elmer LS-55 fluorescence spectrophotometer.

### Entrapment efficiency and loading efficiency

The mixture was centrifuged at 12,500 rpm for 3 min to determine the entrapment efficiency of RVS within MSNPs-CS-DX after the drug loading process. After separating the nanoparticles and liquid phase by centrifuge, the supernatant was removed, and the drug concentration in the supernatant was measured by UV-vis analysis at 252 nm. The entrapment and loading efficiencies were calculated using the following equations that represent the amount of drug encapsulated by the carrier and the loading capacity of the drug loaded per unit weight of the nanoparticle, respectively.

1 $${\rm{EE}}\,( \% )=\displaystyle\frac{{\rm{The}}\; {\rm{total}}\; {\rm{amount}}\; {\rm{of}}\; {\rm{RVS}}\; {\rm{within}}\; {\rm{the}}\; {\rm{MSNPs}}-{\rm{CS}}-{\rm{DX}}}{{\rm{An}}\; {\rm{initial}}\; {\rm{amount}}\; {\rm{of}}\; {\rm{RVS}}\; {\rm{taken}}\; {\rm{for}}\; {\rm{loading}}\; {\rm{studies}}}\times 100$$

2 $${\rm{LE}}\,( \% )=\displaystyle\frac{{\rm{The}}\; {\rm{total}}\; {\rm{amount}}\; {\rm{of}}\; {\rm{RVS}}\; {\rm{entrapped}}\; {\rm{within}}\; {\rm{the}}\; {\rm{MSNPs}}-{\rm{CS}}-{\rm{DX}}}{{\rm{The}}\; {\rm{yield}}\; {\rm{of}}\; {\rm{drug}}-{\rm{loaded}}\; {\rm{NPs}}}\times 100$$

### In vitro drug release studies

An in-vitro evaluation of RVS release from MSNPs-DX-CS and assessing pH-sensitive release using PBS solutions at four different pH levels (4, 5, 7, 10) was conducted. The nanoparticles collected and dried after the loading process were dispersed in fresh PBS solutions. The pH levels of the four PBS solutions were adjusted using buffer solutions. The total duration of in-vitro release was 48 h. The samples were incubated, and at specific time intervals (0.5, 2, 4, 8, 16, 24, 36, 48 h), the solutions were filtered. Concentrations of released RVS were measured in 32 samples using the UV–vis method, with concentrations matched to those observed during the loading step.

### Cell culture and MTT assay

HUVECs in suspension were seeded at a density of 1 × 10^4^ cells per well in a 96-well microtiter plate, achieving approximately 80% confluency. The medium was replaced with a drug-loaded MSNPs and MSNPs-CS-DX suspension medium, with MSNPs concentrations of 10 μg/ml in both cases and RVS concentrations of 1 μg/ml in both cases. The cells were then incubated at 37 °C for 24 and 48 h. The cell culture medium was prepared from Gibco (Germany), with the concentration of fetal bovine serum (FBS) from Pan (Germany) adjusted to 10%. Additionally, the CO2 concentration was maintained at 5%. The drug-loaded medium was added to the wells and incubated for 24 and 48 h. Subsequently, the wells were washed, and the cells were fixed in 5% paraformaldehyde, followed by a final wash with PBS. The cells were then washed twice with PBS, and MTT was added to each well to achieve a final concentration of 0.5 mg/ml. The cells were incubated for 4 h at 37 °C. After MTT incubation, a dimethylsulfoxide solution was used to dissolve the formazan crystals, and the absorbance value at 570 nm was measured for each sample. Each sample was analyzed three times [28, 33].

## Results and discussion

### Synthesis and characterization of the nanoparticles

**Fig. 1:**
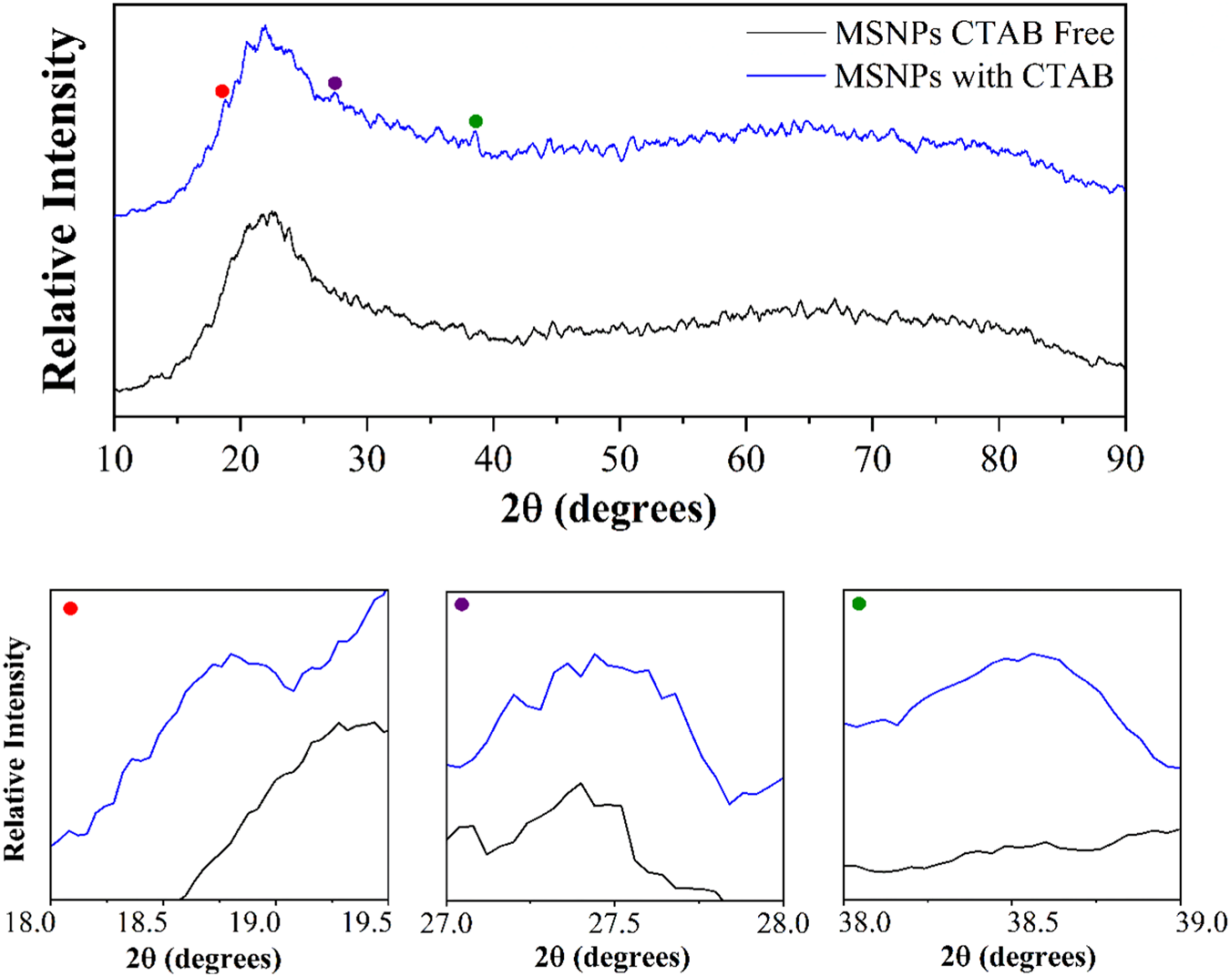
Wide-angle XRD pattern of synthesized mesoporous silica with CTAB and without CTAB

Figure 1 shows the XRD patterns of mesoporous silica with and without CTAB. Both patterns illustrate a peak in the range of 19–24° associated with the mesoporous silica [34–37]. The XRD pattern of mesoporous silica with CTAB indicates three low-intense peaks at 2θ = 18.8, 27.7, and 38.5 [38], which is related to the structure of mesoporous silica with CTAB and disappears when the sample was CTAB-free. This synthesis method is shown to be able to eliminate the CTAB pattern. It is worth noting that using methanol and HCl without any heat treatment which may led to grain coarsening, removing the CTAB pattern, and synthesizing mesoporous silica.

**Fig. 2:**
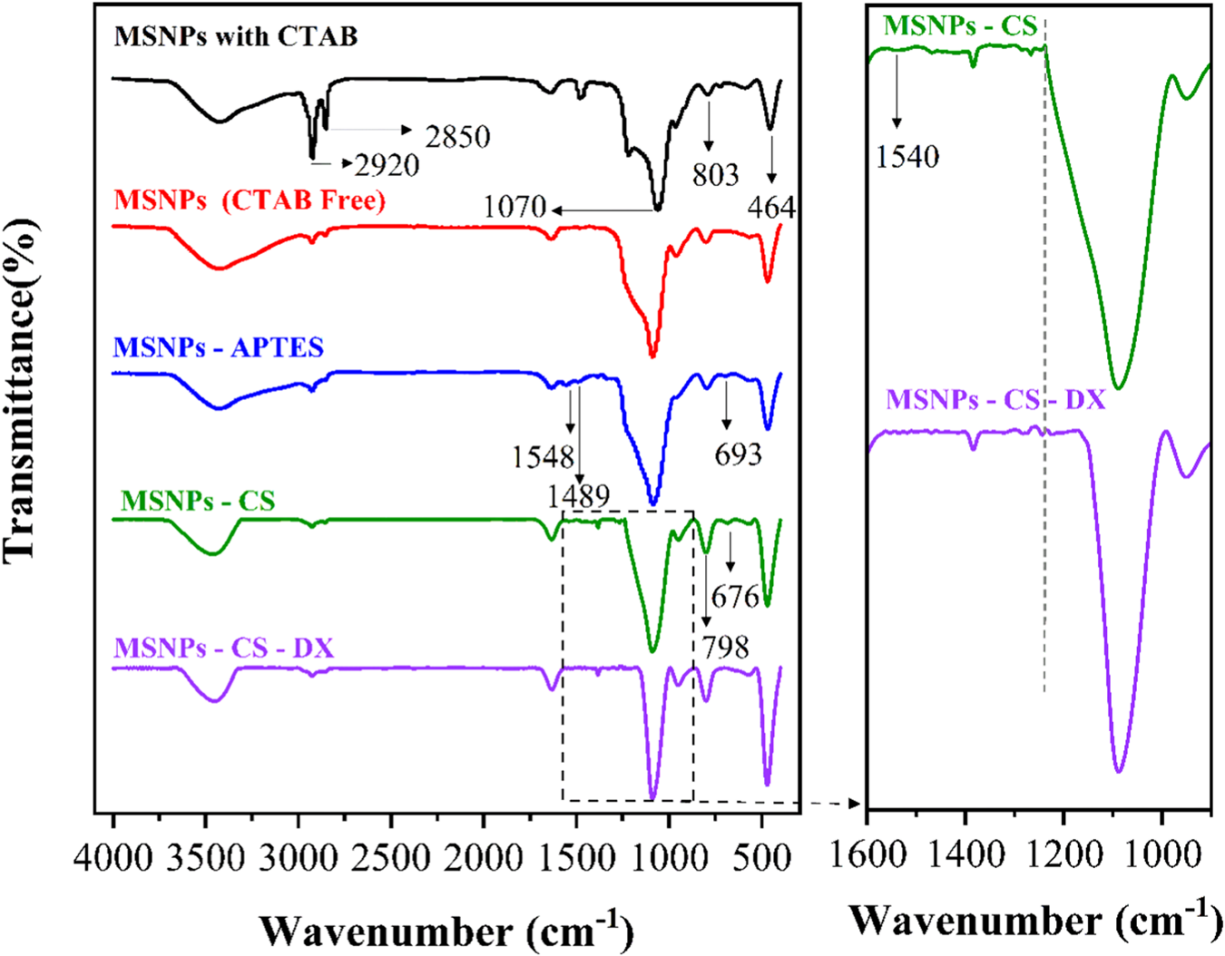
The FTIR patterns of mesoporous silica with and without CTAB, mesoporous silica functionalized with APTES, mesoporous silica with chitosan functionalized with APTES, and mesoporous silica with CS-DX functionalized with APTES

The synthesized pure and layered MSNPs were further evaluated using FTIR. The spectrum of pure MSNPs shows three peaks at 1070, 803, and 464 cm^−1^, representing the Si–O–Si bonding. Two distinct peaks in 2920 and 2850 cm^−1^ are visible, exhibiting C—H bonding in CTAB. The peaks at 2920 and 2850 cm^−1^ almost disappeared in the CTAB-free MCM-41 pattern in Fig. 2, indicating the removal of CTAB from MCM-41 [39, 40]. The peaks at 693 and 1548 cm^−1^ represent the APTES structure. The peak at 1548 cm^−1^ appeared due to the formation of an amine group during the functionalization of mesoporous silica using APTES [41, 42]. Also, two peaks at 1489 cm^−1^ prove the formation of [–NH3^+^ (HCO3)^−^] during the calcination of APTES at room temperature [43]. Two peaks at 676 and 798 cm^−1^ and a low-intense peak at 1540 cm^−1^ indicate N–H bonding existed in the structure of CS [44]. Concerning CS structure, the peak at 900–1250 cm^−1^ in the CS sample ends at the wavenumber of 1247 cm^−1^. This wavenumber was shifted to 1150 cm^−1^ in the dextran sulfate sample. This change was related to the formation of sulfate groups by crosslinking between positively charged ions [45]. It can be concluded that the layered structure of CS-DX sulfate was formed on the surface of mesoporous silica.

**Fig. 3:**
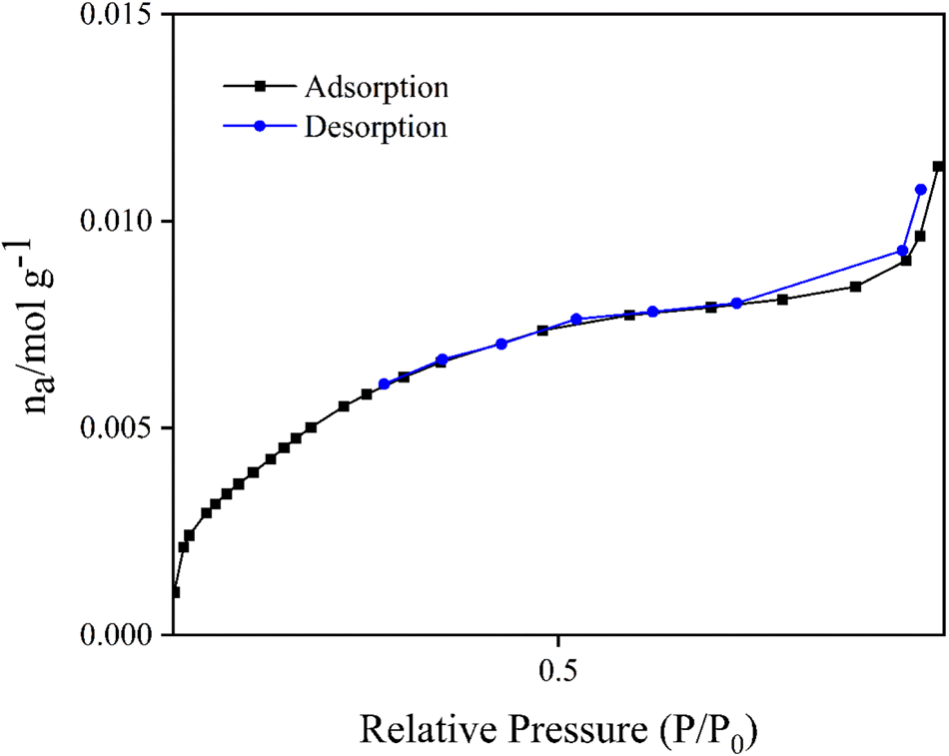
The results of the adsorption/desorption BET test of MSNPs with APTES

The MSNPs with APTES were characterized by BET nitrogen adsorption-desorption measurements to assess pore size, porosity, and active surface area. Figure 3 shows a typical IV-type curve of mesoporous material with a specific surface area of 439.79 m^2^/g, average pore diameter, and pore volume of 3.5 nm and 0.38 cm^3^/g, respectively. The finding is consistent with previous studies on the BET curves [35, 46].

### Characterization of the pure and layered MSNPs particles

**Fig. 4:**
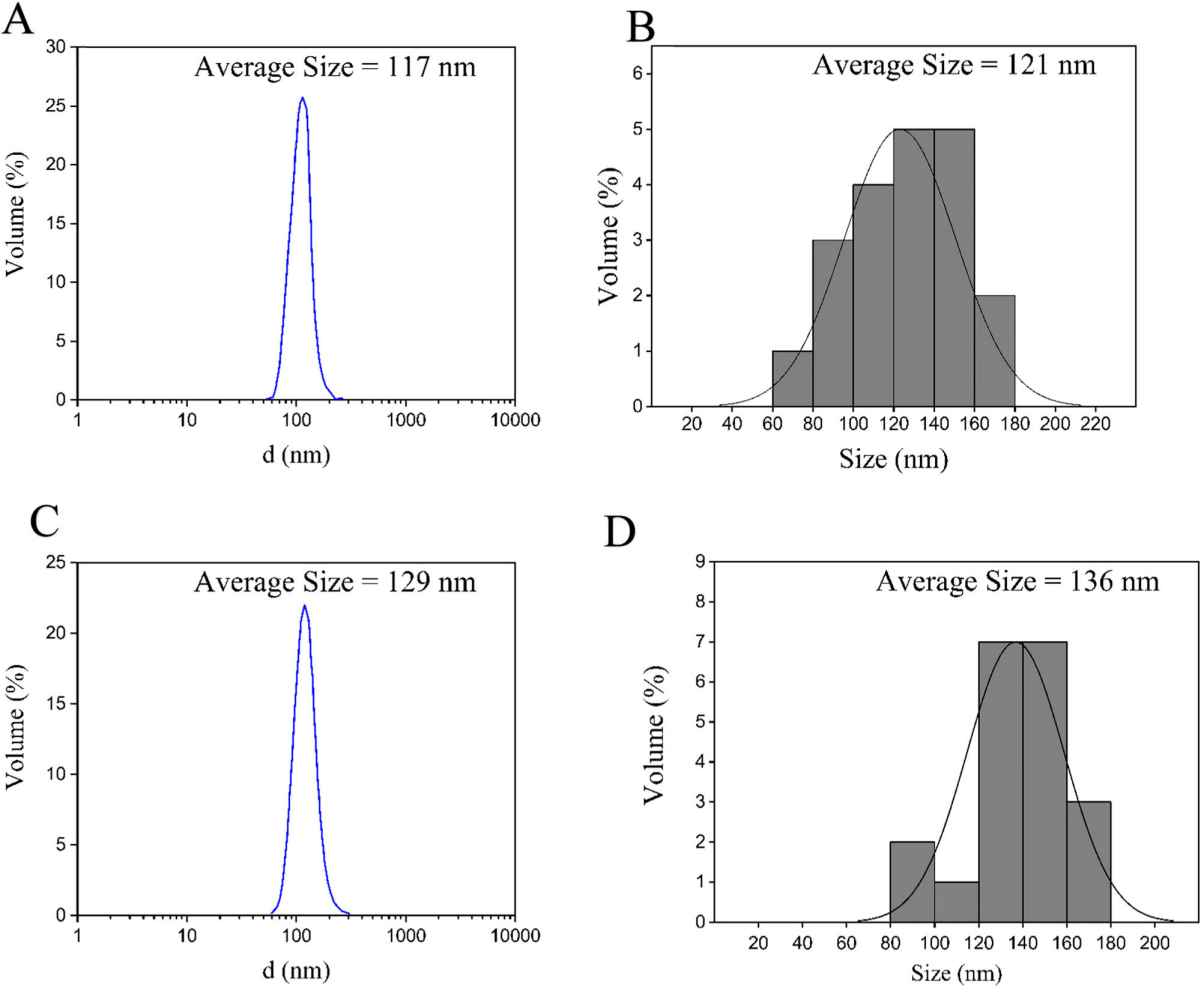
The results of DLS analysis (**A**) and (**B**) MSNPs, (**C**) and (**D**) MSNPs-CS-DX, (**A**) and (**C**) show the average diameters of particles, and (**B**) and (**D**) show the most repetitive particle size

The results of pure and multilayer MSNPs particle size measured by DLS are illustrated in Fig. 4. According to Fig. 4a, c, the average particle diameters of pure MSNPs and MSNPs-CS-DX are about 117 and 129, respectively. Figure 4B demonstrates that the particle size of MSNPs lies in the range of 60–180 nm, while based on Fig. 4D, in the case of MSNPs-CS-DX, this value fluctuates from 80 to 180 nm. The average diameter and particle size of layered MSNPs are about 15 nm larger than that of pure MSNPs due to the formation of different layers on the surface of mesoporous particles. Nanocarriers circulating in normal blood vessels do not easily leave the capillaries that flow in the tissues such as the kidney, lung, and heart if they have a diameter range of 100–150 nm. On the other hand, smaller particles in the size range of 20–100 nm may spread to bone marrow, spleen, and liver and also may relinquish the bloodstream via the drilled capillaries of these organs to some degree [47]. Therefore, it can be concluded that the size of the resulting nanoparticles in the present work is in the ideal range for drug delivery.

**Fig. 5:**
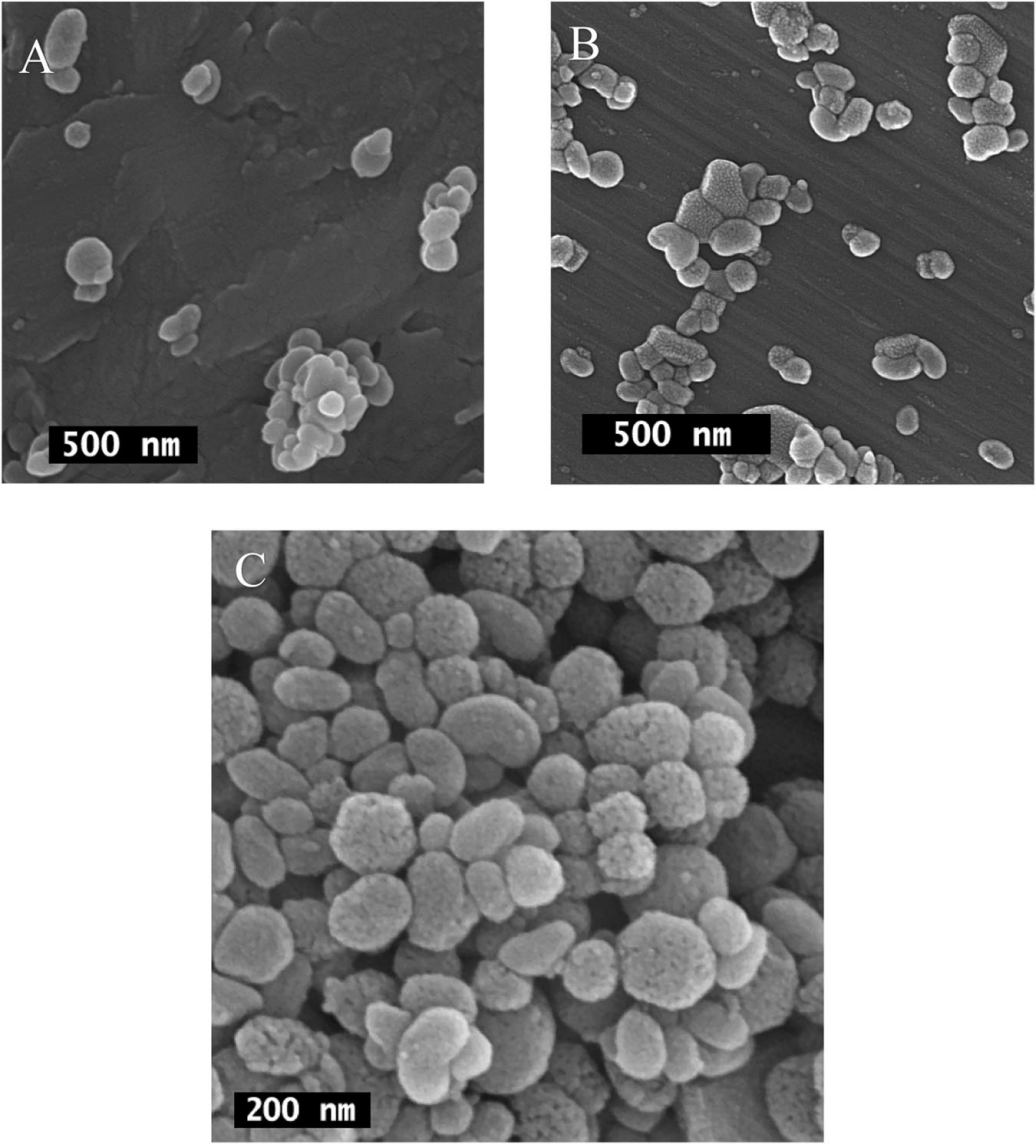
The FE-SEM images of (**A**) pure MSNPs, (**B**) MSNPs-CS-DX particles, and (**C**) MSNPs-CS-DX

Figure 5 demonstrates the FE-SEM images of pure and layered MSNPs. The pure MSNPs particles have spherical morphology with a smooth surface and an average size of 125 nm (Fig. 5A). Figure 5B indicates that the smooth surface has a spongy structure in layered MSNPs. The average particle size of layered MSNPs is around 135 nm. These results are in agreement with the results of the DLS experiment. The MSNPs-CS-DX particles can be observed in Fig. 5C in larger magnification. The image shows the spongy structure of the particles’ surface due to the formation of the CS layer.

**Fig. 6:**
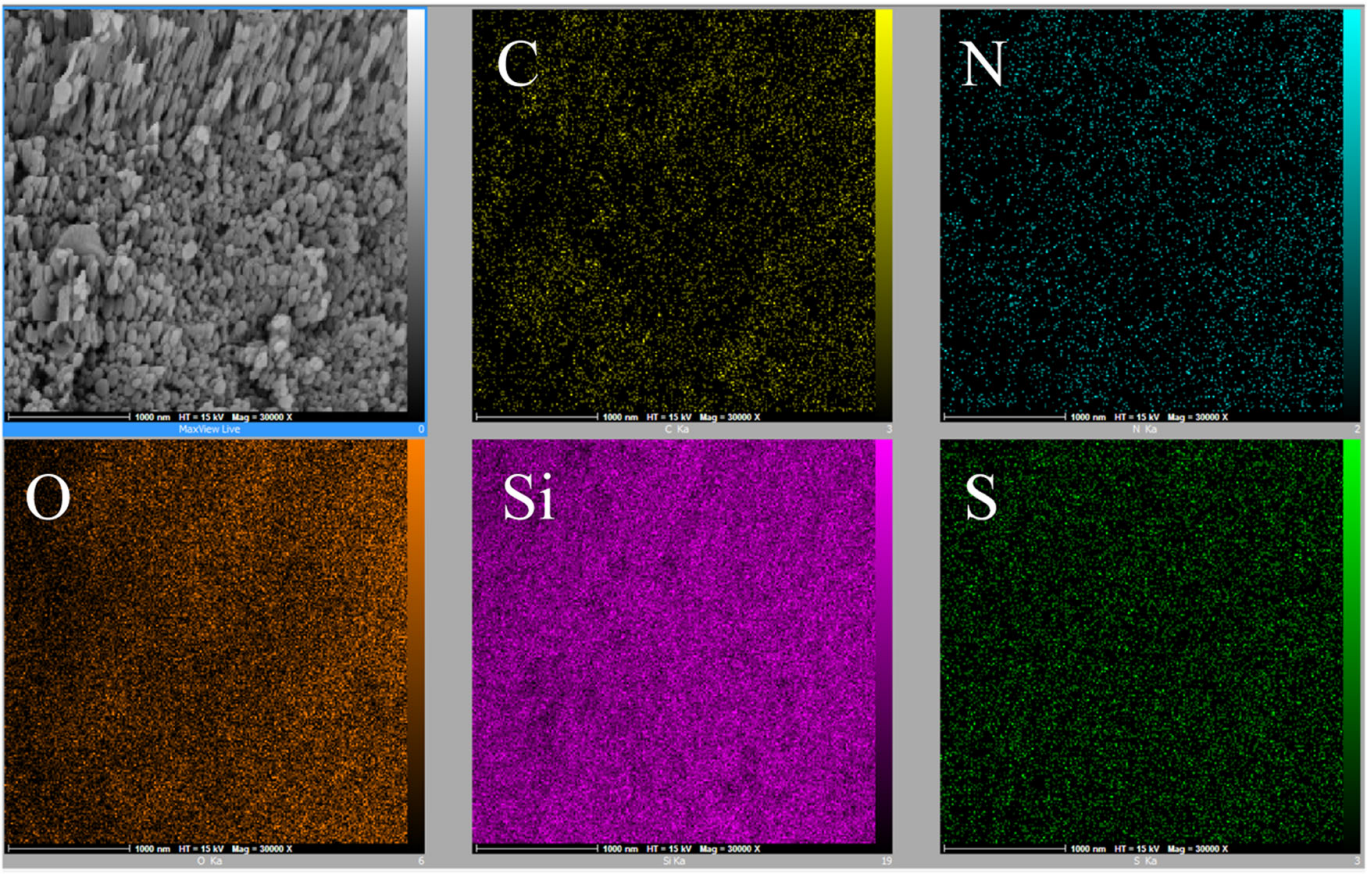
EDS map analysis of various elements in MSNPs-CS-DX: The top row from left to right related to map target, distribution of Carbon, and Nitrogen. The bottom row from left to right related to Oxygen, Silicon, and Sulfur

Figure 6 shows the EDS analysis of the MSNPs-CS-DX sample, performed to evaluate the distribution manner of the elements in mesoporous silica, CS, and dextran sulfate. Concerning the amount of Si in the image, It can be stated that the high, uniform, and desirable distribution of Si in the image is due to the mesoporous silica core of the carrier.

The overlapping of oxygen and silicon distribution indicates the presence of silica; similarly, the uniform distribution of carbon and nitrogen proves the formation of an organic CS network. The presence of dextran sulfate is shown in the elemental map by the uniform distribution of the sulfur.

**Fig. 7:**
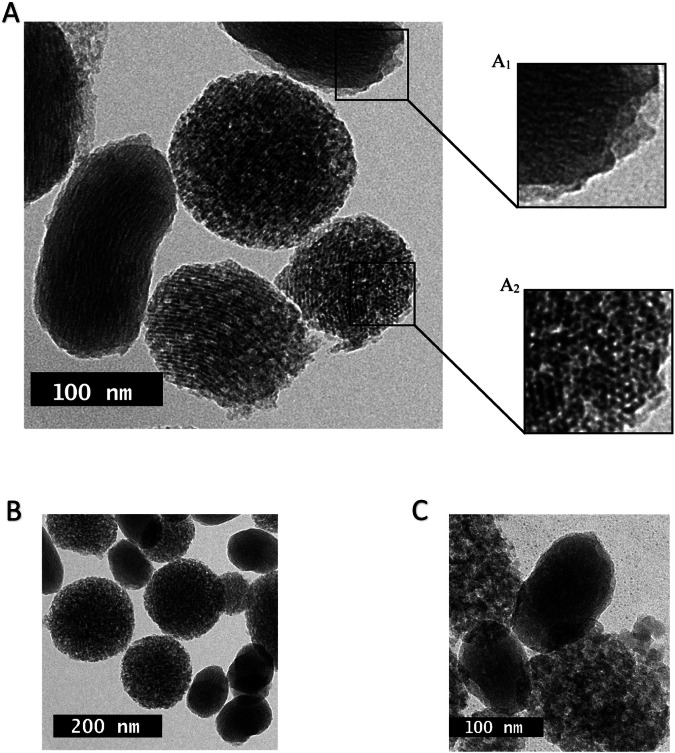
TEM image of (**A**) the PEC layer coated MSNPs, (**A**_**1**_) a particle with PEC layer, (**A**_**2**_) the porous structure of MSNPs, (**B**) a single particle without PEC layer, and (**C**) the PEC layer coated MSNPs

Figure 7 shows the TEM images of MSNPs, polyelectrolyte complexes (PEC), and the PEC-coated MSNPs. As shown in Fig. 7A1, the PEC layer consisting of CS and DX is formed as a halo around the mesoporous silica core, demonstrating a uniform distribution with an average thickness of about 10–15 nm. According to Fig. 7A2, MSNPs are porous and have a honeycomb-like structure. Pristine MSNPs shown in Fig. 7B demonstrate spherical particles without any halo, while the PEC-coated MSNPs illustrate a halo, showing the formation of PEC on their outer surface.

### Loading efficiency and in vitro drug release studies

**Fig. 8:**
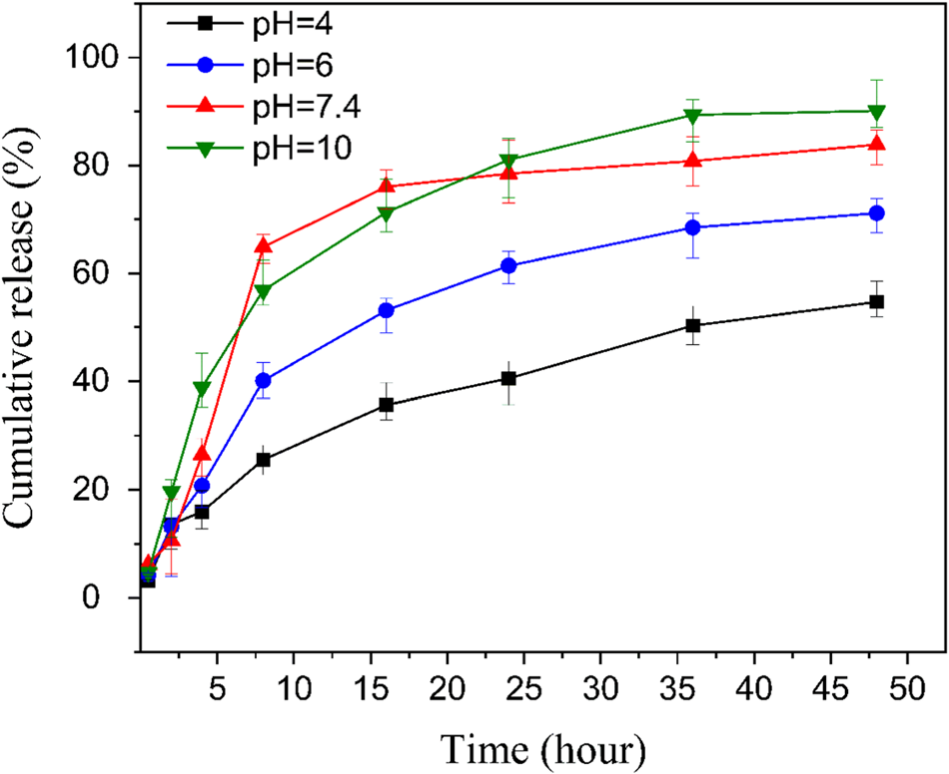
In-vitro release curve of MSNPs-CS-DX-RVS at four different pHs of 4, 6, 7.4, and 10

According to the equation described in the experimental section, entrapment and loading efficiencies of MSNPs-CS-DX were calculated at 57 and 13.6%, respectively. Typically, a loading efficiency of around 13% is considered suitable for MSNPs incorporating APTES, CS, and other layers in similar studies. For instance, in an investigation by Shakeran et al., an approximate loading efficiency of 13% was achieved for mesoporous silica-APTES-CS-methotrexate in various loading environments [48]. In this context, Liao et al., synthesized mesoporous silica with CS, achieving an encapsulation efficiency of 35.6% (app), confirming our outstanding 57% EE in this experiment [49]. In-vitro drug release studies were done via the direct dispersion method at pH 4, 6, 7.4, and 10. Figure 8 shows the drug release curve of MSNPs-CS-DX. According to the results obtained from the in-vitro drug release curve in various pHs, the burst release is controlled by CS when the pH is equal to four. The acidic environment causes the CS amino group to hold a more positive charge, being protonated from –NH_2_ to –NH_3_+. The presence of –NH_3_+ in the composition increases the attraction between carboxyl groups in RVS in the interaction and CS. Furthermore, with CS being more positively charged, the electrostatic charge between CS and DX increases as well [50]. The alteration of the particles’ charge has led to a situation where burst release has not occurred in acidic conditions. Similarly, when pH is equal to six, the electrostatic force between CS and DX, and also RVS controls the release kinetics [51]. Although the positive charge reduces compared to pH equal to four, the burst release does not occur, mainly due to the electrostatic charge. However, it should be noted that the amount of drug release is increased compared to the situation where the pH was fixed at 4. Ultimately, as pH equals 7.4, the attraction force between CS, RVS, and DX no longer exists. As a result, the release follows its natural course, and the burst release becomes visible. Regarding alkaline environments, the polymer shell of CS collapses and acts as a protective layer around the mesoporous silica, intercepting the burst release. Dang et al., [52–54]. This is the mechanism that controls the release process in the alkaline environment. While in the acidic medium, the electrostatic force between the components is the controlling mechanism of the release. The protective and condensed layer of CS acts as a preventive factor against burst release. As can be seen in the initial 8 h of release in the curve related to pH = 4, 6, 7.4, 10, the drug release is 25, 40, 57, and 64%, respectively. The drug release at pH = 4, 6, 7.4, and 10 reaches 40, 61, 78, and 81% after 24 h, respectively. Based on the mentioned discussion, it is axiomatic that the designed nanocarriers are sensitive to pH. It also seems that depending on the pH value, two mechanisms prevail in drug release kinetics.

### In vitro cytotoxicity by MTT assay

**Fig. 9:**
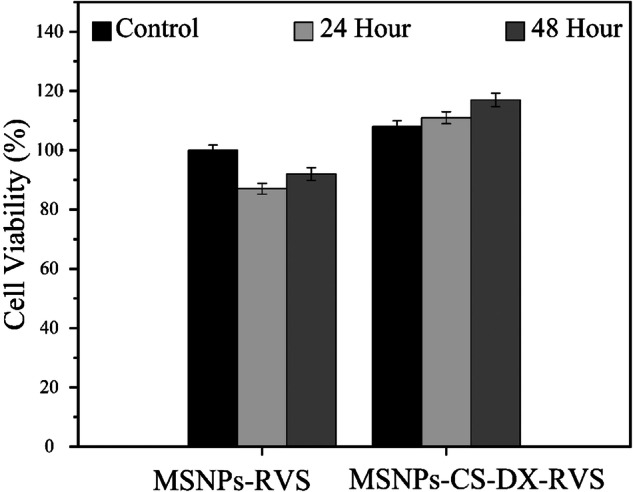
The result of MTT assay in 24 h and 48 h for MSNPs-RVS and MSNPs-CS-DX-RVS, the concentration of MSNPs and MSNPs-CS-DX = 10 μg/ml, the concentration of RVS = 1 μg/ml

The toxicity of MSNPs-RVS and MSNPs-CS-DX-RVS was analyzed by MTT assay. This test was performed during two sleep times of 24 and 48 h. As seen in Fig. 9, when RVS-carrying MSNPs were cultured together with HUVEC cells, the survival of seeded cells decreased relatively little during 24 h of sleep. It is observed that they reduced by 13% compared to the control sample. The reduction in cell survival compared to the control sample was decreased to 8% at 48 h, which is less than at 24 h interval. In the 24 h sleep period, MSNPs-CS-DX-RVS showed a 24% increase compared to the same period for MSNPs-RVS. This value comes to 17% compared with two samples in 48 h, which is a promising result. This increase can be due to normal cell division. Comparing two control samples in two cases where MSNPs alone and MSNPs-CS-DX are drug carriers, CS and DX showed 9% better survival for the control sample. The samples containing CS and DX also helped to increase the cell viability at each stage during the 24 and 48 h sleep periods. To compare MTT assay results, we evaluate some case studies for comparison with our results. First, Zhang et al., conducted an MTT assay on Polyethylenimine-Modified Mesoporous Silica using Huve cells. The data revealed that polyethyleneimine-modified MSNs decreased HUVEC cell viability compared to bare mesoporous silica. This effect was exacerbated by the addition of ATG5 siRNA to polyethylenimine-modified MSNs [55]. Secondly, Song et al., demonstrated that adding simvastatin as a drug to Hyaluronic Acid-Functionalized Mesoporous Silica Nanoparticles decreased HUVEC cell viability [56]. However, this effect did not apply to RVS. Finally, Barros et al., showed that adding hydroxyapatite to mesoporous silica had no meaningful influence on HUVEC cell viability [57]. However, this study shows that the CS and DX layers not only have no toxicity to the cardiovascular system but also help decrease toxicity and increase biocompatibility.”

## Conclusions

In this study, MSNPs were coated with PEC comprised of CS and dextran sulfate (DX). The synthesis method was a single-step process utilizing a layer-by-layer approach, providing a streamlined route to avoid multi-step processing. Using methanol and HCL, the CTAB pattern was diminished without heat treatment. FTIR pattern also proved CTAB removal and showed the formation of CS-DX coating. DLS measurement demonstrated that the mean particle size of the layered mesopores was 129 nm, with an average diameter of the CS-DX layer of 10–15 nm. Spherical grains with the optimal particle size distribution of 80–160 nm were satisfactory for the PEC sample. These results were shown to be promising for drug delivery purposes. Also, the results obtained from DLS were proved by SEM images. Due to the presence of mesoporous silica, as the pore size and porosity obtained from BET evaluation were satisfying, the drug delivery system was designed to encapsulate the efficiency of more than 57% of the drug. It was shown that MSNPs-CS-DX was pH sensitive, mainly due to the existence of the amino group of CS at different pH and its effect on the electrostatic bond. The in-vitro drug release curve demonstrated that the drug release kinetics was controlled at pH = 4 and 6 while increasing pH caused the burst release. The pH sensitivity is reported to be necessary for drug delivery application. The toxicity of the nanometer system carrying the drug decreased with the formation of PEC confirmed in all three control samples after 24 and 48 h of sleep. In addition to the mentioned features, the synthesized nanometer system may lead to promising results in the treatment of heart diseases as well as preventing the formation of new plaques and disrupting the accumulation process due to the properties of CS and DX. In sum, MSNPs-CS-DX was synthesized for the first time using a single-step synthesized route, showing descent performance for drug delivery applications due to its pH sensitivity and non-toxicity that arose from the presence of DX.

## Data Availability

Data will be made available on request.
